# Genetic diversity of canine olfactory receptors

**DOI:** 10.1186/1471-2164-10-21

**Published:** 2009-01-14

**Authors:** Stéphanie Robin, Sandrine Tacher, Maud Rimbault, Amaury Vaysse, Stéphane Dréano, Catherine André, Christophe Hitte, Francis Galibert

**Affiliations:** 1 Institut de Génétique et Développement de Rennes, CNRS UMR 6061, Université de Rennes 1, 2 Avenue du Professeur Léon Bernard, CS 34317, 35043 Rennes, France; 2 Systèmes d'Elevage Nutrition Animale et Humaine, INRA UMR 1079, Domaine de la Prise, 35590 Saint Gilles, France

## Abstract

**Background:**

Evolution has resulted in large repertoires of olfactory receptor (OR) genes, forming the largest gene families in mammalian genomes. Knowledge of the genetic diversity of olfactory receptors is essential if we are to understand the differences in olfactory sensory capability between individuals. Canine breeds constitute an attractive model system for such investigations.

**Results:**

We sequenced 109 OR genes considered representative of the whole OR canine repertoire, which consists of more than 800 genes, in a cohort of 48 dogs of six different breeds. SNP frequency showed the overall level of polymorphism to be high. However, the distribution of SNP was highly heterogeneous among OR genes. More than 50% of OR genes were found to harbour a large number of SNP, whereas the rest were devoid of SNP or only slightly polymorphic. Heterogeneity was also observed across breeds, with 25% of the SNP breed-specific. Linkage disequilibrium within OR genes and OR clusters suggested a gene conversion process, consistent with a mean level of polymorphism higher than that observed for introns and intergenic sequences. A large proportion (47%) of SNP induced amino-acid changes and the Ka/Ks ratio calculated for all alleles with a complete ORF indicated a low selective constraint with respect to the high level of redundancy of the olfactory combinatory code and an ongoing pseudogenisation process, which affects dog breeds differently.

**Conclusion:**

Our demonstration of a high overall level of polymorphism, likely to modify the ligand-binding capacity of receptors distributed differently within the six breeds tested, is the first step towards understanding why Labrador Retrievers and German Shepherd Dogs have a much greater potential for use as sniffer dogs than Pekingese dogs or Greyhounds. Furthermore, the heterogeneity in OR polymorphism observed raises questions as to why, in a context in which most OR genes are highly polymorphic, a subset of these genes is not? This phenomenon may be related to the nature of their ligands and their importance in everyday life.

## Background

Olfactory receptors (OR) are expressed on the cilial membranes of olfactory sensory neurons embedded in the olfactory mucosa [1-3]. OR are transmembrane G-protein-coupled receptors and constitute the first element in a biochemical cascade leading to the perception and recognition of an odorant. OR genes constitute the largest mammalian gene family, with several hundred genes in the human genome and up to 1550 in the rat genome [4-8]. Comparisons of the amino-acid sequences deduced from orthologous and paralogous OR genes have shown a large number of positions to be highly conserved and others to be variable. The conserved residues are thought to be involved in signal transduction, whereas the variable residues are thought to be involved in binding thousands of odorant molecules in specific interactions [7,9-11].

Mammals have evolved sophisticated systems for sensing the outside world and, in particular, for sensing odorant molecules indicating danger or the presence of a mate or food. Dogs are particularly interesting in this respect. They were domesticated from wolves some 15,000 years ago and have since undergone extensive breeding and selection, resulting in 400 or so different breeds, some of which were developed specifically for hunting, in which olfaction plays a central role [12-15]. The astounding ability of dogs to detect an odorant molecule and follow its trace results from the interaction of several brain functions. The first step in this process involves the efficient binding of an odorant molecule to a given set of OR. The absence of a particular OR or the presence of alleles giving rise to OR with a low binding efficiency would lead to poor downstream processing or the complete absence of such processing. As a case in point, links between nucleotide polymorphisms in two OR genes in humans (OR7D4 and OR11H7P) and the perception of specific odorants – androstenone and isovaleric acid, respectively – have recently been demonstrated [16-18].

We therefore wondered whether breeds or individual dogs known to be particularly skilled at odorant detection have different gene alleles encoding OR with a higher affinity for their ligands or more efficient at initiating the signal transduction cascade. In a preliminary study on a subset of 16 OR genes, we showed the rate of polymorphism to be high, with all genes having at least one SNP in their open reading frame (ORF) [19]. This finding led us to analyse the DNA sequences of a larger number of OR genes (109 OR genes) in a cohort of 48 dogs from six breeds known to differ in their ability to detect odorants: four breeds known for their strong sense of olfaction (German Shepherd, Belgian Malinois, English Springer Spaniel, and Labrador Retriever) and two breeds known to have a weak sense of olfaction (Greyhound and Pekingese).

We show here that OR genes are generally highly polymorphic, with a mean of one SNP per 577 nucleotides. However, the degree of polymorphism observed is highly variable, with some OR genes having few if any SNP and others being highly polymorphic (1 SNP/122 nt). This high level of genetic polymorphism, resulting in a large number of amino-acid substitutions in all parts of the OR, strongly suggests that a large proportion of the mutations occurring during DNA replication are not counter-selected, facilitating the evolution of the OR repertoire and increasing its potential to recognise odorants.

## Methods

### DNA samples

DNA was obtained from 48 dogs from six breeds: German Shepherd Dog (GSD), Belgian Malinois (BM), Labrador Retriever (LR), English Springer Spaniel (ESS), Greyhound (Grey), Pekingese (Pek). In addition, blood samples from 8 Boxer (Box) dogs were processed for the analysis of a subset of OR genes.

Most of the DNA samples were obtained from the caniDNA bank [20] and were selected from dogs with no family links up to grandparental level. We also selected dogs from different breeders from different regions or countries, to minimise possible links between animals. When necessary, the panel was completed with additional samples provided by Gary S. Johnson (Department of Veterinary Pathobiology-University of Missouri, USA) and Paul G. Jones from Masterfoods (England).

DNA was extracted with the Nucleospin Blood L kit (Macherey Nagel). For samples with low DNA concentrations, whole genome amplification was carried out with the Illustra GenomiPhi V2 DNA Amplification Kit (GE Healthcare).

### PCR amplification and OR gene sequencing

Pairs of specific primers (20–30 bp) were designed with Primer3 [21], for binding outside the reading frame, for amplification of the whole OR ORF. Primers were also designed to bind to regions with a unique sequence, to ensure that paralogous genes were not amplified. The nomenclature and sequences of OR genes were extracted from the paper by Quignon *et al*. [7] and can be obtained from [22]. PCR amplification was carried out in a final volume of 10 μl, containing 35 ng of dog DNA, GeneAmp 1 × PCR Gold Buffer, 2 mM MgCl_2 _(Applied Biosystems), 250 μM dNTP (GE Healthcare), 0.5 U AmpliTaq Gold DNA Polymerase (Applied Biosystems) and 0.3 μM of each specific primer. PCR conditions were as follows: initial denaturation at 95°C for 7 min, 20 cycles of 94°C for 30 s, 61°C for 30 s with a touch-down process (-0.5°C per cycle) and 72°C for 1 min, 15 cycles of 94°C for 30 s, 51°C for 30 s, 72°C for 1 min, and a final extension at 72°C for 3 min. Aliquots of PCR products were subjected to electrophoresis in 1% agarose gels in 0.5 × TBE. We then purified 2.5 μl of PCR products from faithful amplifications using 1 μl of ExoSAP-IT (USB). The purified PCR products were incubated at 37°C for 15 min and then at 80°C for 15 min. Pairs of specific internal primers (18–21 bp) designed with Primer3 [21] were used for sequencing PCR products with the BigDye V3.1 Cycle Sequencing Kit (Applied Biosystems), used according to the manufacturer's instructions. Sequencing products were fractionated on a 3130xl genetic analyser from Applied Biosystems.

### SNP identification

Sequences were aligned and analysed with SeqScape software V2.5 (Applied Biosystems), using the CanFam2 DNA sequence as a reference [23]. Only SNP corresponding to nucleotide sequence of the highest quality, as determined by the Phred algorithm [24], were retained.

### Data analysis

Haploview software v4.0 [25] was used to calculate the SNP MAF (minor allele frequency) and LD values. We calculated r^2 ^values for OR genes and D' values for clusters, making it possible to compare our results with those of previous studies [23,26].

### Haplotypes

Haplotypes were inferred using fastPHASE software v 1.0.1 with the default settings [27]. This software estimates the missing genotypes and reconstructs haplotypes from unphased genotype data from unrelated individuals.

### N value calculation

As an index of the level of OR polymorphism, a mean distance N between SNP was calculated, based on the number of SNP detected through the pairwise comparison of all OR sequences and the occurrence of the two alleles of each SNP. Thus, the smallest N value denotes the highest level of polymorphism.

The N value for individual OR genes was calculated as follows:

$${\text{N}}_{\text{OR}}=(\text{ORF size}\times \text{pairwise comparison})/\sum_{i=1}^{n}{\text{a}}_{\text{i}}\cdot {\text{b}}_{\text{i}}$$

where n is the number of SNP per OR gene and a and b the occurrences of the two alleles.

The N value for the complete set of OR genes was calculated with the same formula, in which n is the total number of SNP and the individual ORF size is replaced by the sum of individual ORF sizes.

### Ka/Ks

Ka/Ks was calculated for each OR gene, as described by Goldman and Yang [28], using the CODEML program (model = 0) from the PAML package [29]. Ka/Ks for the whole set of OR genes was obtained by determining mean Ka/mean Ks.

## Results and Discussion

### SNP number and distribution

We analysed the nucleotide sequences of 109 OR genes (102 genes and seven pseudogenes, as defined in the genome sequence [23]) selected from the entire OR repertoire of 872 genes and 222 pseudogenes [7,30]. These OR genes were selected to be representative of a large number of families (the five class I families and 15 of the 18 class II families), subfamilies and clusters (33 of 54) located on 20 chromosomes (Additional file 1). They were also selected as representative of genomic regions very rich in OR genes, as for cluster @40–44 on canine chromosome 18 (CFA18), or with a lower density of OR genes, as for cluster @3 on CFA15. We also studied five isolated OR genes. We determined the nucleotide sequences of PCR fragments amplified from DNA purified from a cohort of 48 dogs of six breeds: German Shepherd Dog (GSD), Belgian Malinois (BM), Labrador Retriever (LR), English Springer Spaniel (ESS), Greyhound (Grey) and Pekingese (Pek). We also analysed a subset of 27 OR genes in eight Boxers (Box).

**Figure 1:**
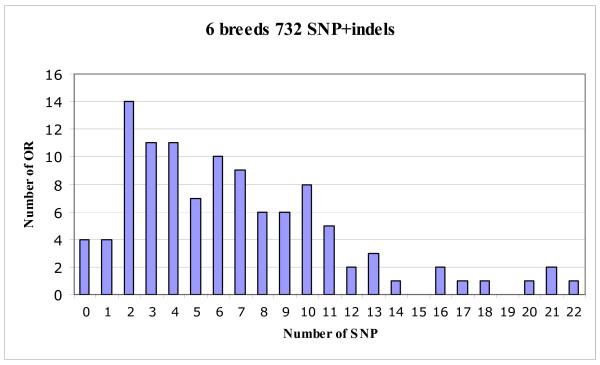
**Distribution profile of the 732 SNP + indels**.

Visual inspection of all sequencing traces obtained with the cohort of 48 dogs led to the identification of 710 SNP, corresponding to 549 transitions and 161 transversions. We also observed 17 short insertions/deletions (indels, 1 to 3 nt) and five longer indels of 6 to 74 nucleotides. As the occurrence of each indel probably corresponded to a single mutational event, these 732 mutations (SNP + indels) were combined for further analysis. Figure 1 shows the distribution of SNP within the 109 OR genes. It shows that all but four of the OR genes are polymorphic, with one to 22 SNP per OR gene.

**Figure 2:**
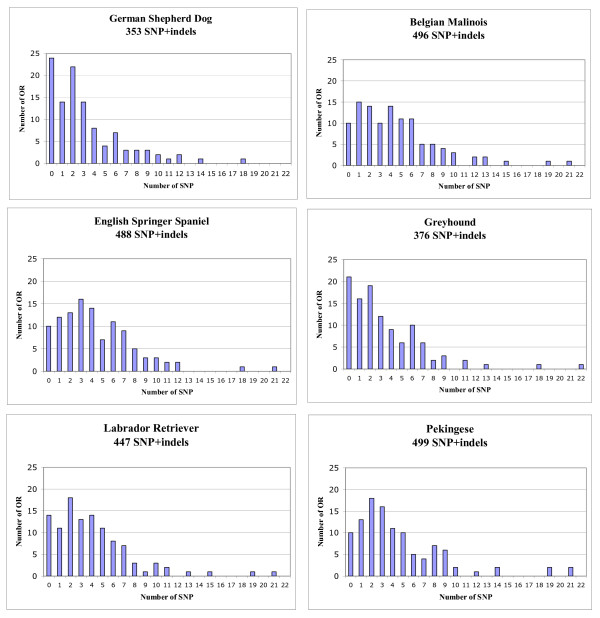
**Distribution of SNP within the 6 breeds**.

When analysed at the breed level, the total number of SNP differed significantly (chi^2^, P < 10^-3^) between breeds, whereas their distribution did not (Wilcoxon-Mann-Whitney) (Figure 2). However the numbers of OR genes without SNP differed markedly between breeds (chi^2^, P < 0.05), with 24 and 21 OR genes with no SNP for German Shepherd Dog and Greyhound, respectively, 14 for Labrador Retriever and only 10 for each of the three other breeds. The set of OR genes with no SNP was either breed-specific or shared by only a few breeds, in different combinations (Table 1).

**Table 1:** OR genes with no SNP in one or several breeds.

**OR name**	**GSD**	**BM**	**ESS**	**Grey**	**LR**	**Pek**	**Breeds number without SNP**
**CfOR16F03**	0	0	0	0	0	0	6
**CfOR0154**	0	0	0	0	0	0	6
**CfOR0166**	0	0	0	0	0	0	6
**CfOR0317**	0	0	0	0	0	0	6
**CfOR0606**	0	0	0	0	0	1	5
**CfOR08C09**	0	0	0	0	0	1	5
**CfOR0390**	0	0	0	6	0	1	4
**CfOR08A02**	0	6	0	0	0	10	4
**CfOR3109**	0	0	1	2	2	0	3
**CfOR0525**	0	2	1	0	1	0	3
**CfOR0333**	1	1	0	0	0	1	3
**CfOR0064**	0	1	1	0	1	2	2
**CfOR04C07**	0	1	2	0	1	1	2
**CfOR0401**	0	1	4	0	1	2	2
**CfOR04A02**	0	2	2	0	2	2	2
**CfOR0031**	1	0	2	1	0	2	2
**CfOR0050**	1	1	2	0	1	0	2
**CfOR1697**	2	2	0	0	2	2	2
**CfOR08G01**	2	2	2	0	1	0	2
**CfOR04B06**	0	1	2	1	2	1	1
**CfOR0568**	0	1	5	2	2	3	1
**DTPRH02**	0	2	2	2	2	2	1
**CfOR1573**	0	2	2	2	2	2	1
**CfOR2510**	0	4	3	2	5	3	1
**CfOR04C05**	0	4	7	1	4	19	1
**CfOR0130**	0	6	6	6	6	6	1
**CfOR16D10**	0	7	5	5	7	5	1
**CfOR0276**	0	8	7	7	7	8	1
**CfOR0006**	0	13	12	3	15	14	1
**CfOR0173**	1	1	1	0	1	1	1
**CfOR0426**	1	1	1	1	0	2	1
**CfOR0297**	2	1	1	0	2	1	1
**CfOR08A12**	2	2	2	0	2	1	1
**CfOR12G07**	3	2	2	2	0	4	1
**TPCR62**	3	2	3	1	0	2	1
**CfOR0438**	4	0	4	2	4	3	1
**CfOR0149**	4	3	3	2	3	0	1
**CfOR0058**	5	5	5	5	0	5	1
**CfOR0407**	5	6	3	0	6	3	1
**CfOR0043**	6	6	8	0	7	7	1
**CfOR0527**	7	7	8	7	7	0	1

Numbers (0 to 19) refer to the number of SNP per OR gene and per breed. This table also highlights the range of polymorphism of certain OR genes within the six breeds (see, for example, CfOR0006 or CfOR04C05). This table is a subset of additional file 2.

At the whole-population level, most OR genes tended to be either weakly (such as CfOR2171 and CfOR08C09 with 0 or one SNP per breed) or highly (such as CfOR0007 with 18 or 19 SNP and CfOR0034 with 14 to 22 SNP depending on breed) polymorphic (see additional file 2). However, there were several notable exceptions, with some OR genes weakly polymorphic or not polymorphic in one breed and highly polymorphic in the other five breeds. This was the case for CfOR0527 (no SNP in Pekingese but seven or eight SNP in each of the other five breeds), CfOR0390 (six SNP in Greyhound, one SNP in Pekingese and none in the other breeds) and CfOR08A02 (10 SNP in Pekingese, six SNP in Belgian Malinois and no SNP in the other breeds; Table 1).

We investigated the possible correlation between OR gene polymorphism and the organization of these OR genes into clusters of different sizes, by ranking the 109 OR genes according to SNP content. We selected the 22 OR genes with no more than two SNP and the 27 OR genes with 10 or more SNP and compared the sizes of the clusters harbouring these OR genes. As shown in Figures 3.1 and 3.2, the least polymorphic OR genes were preferentially localised in small clusters (median cluster size 4.5 OR genes) and the highly polymorphic OR genes, in large clusters (median cluster size 240 OR genes). Mann-Whitney test showed this relationship to be significant (P < 10^-3^). In addition, the 109 OR genes were ranked according to cluster size and we selected the 20 OR genes located in clusters containing five or fewer OR genes and the 18 OR genes present in the largest cluster (containing 243 OR genes). Again, OR genes in small clusters tended to be less polymorphic than OR genes in large clusters (median SNP numbers of 2 and 8 for the smallest and largest clusters, respectively, Mann-Whitney test; P < 10^-3^) (Figures 3.3 and 3.4). Interestingly, the OR genes with the highest number of SNP tended to have paralogous genes with higher sequence homology (> 90%) than OR genes devoid of SNP or harbouring a small number of SNP.

**Figure 3:**
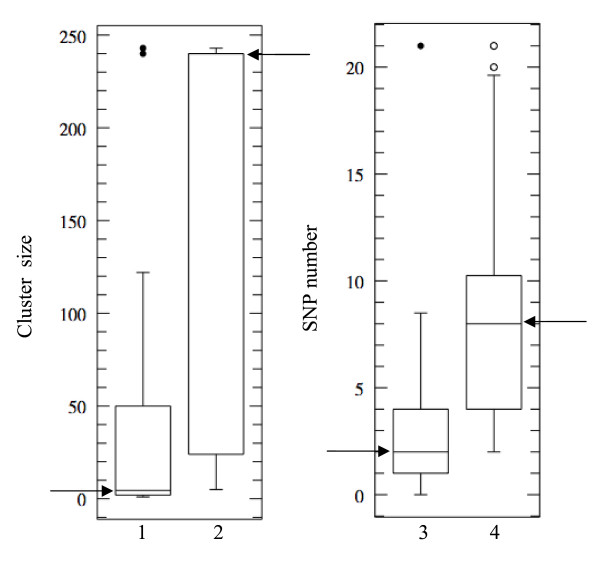
**Boxplot of cluster sizes (1, 2) and boxplot of SNP contents (3, 4)**. Boxplot 1 shows the cluster sizes of the 22 least polymorphic OR genes (≤ 2 SNP). This boxplot should be compared with boxplot 2, showing the cluster sizes of the 27 OR genes with the largest number of SNP (≥ 10 SNP). Boxplot 3 corresponds to the SNP contents of 20 OR genes located in clusters with up to five OR genes. It should be compared with boxplot 4, corresponding to the 18 OR genes located in the largest cluster (243 OR genes). Arrows indicate the median values in the four boxplots.

### Allele frequency

SNP minor allele frequency (MAF) ranged from 1% to 50% (see additional file 3). However, MAF within breeds might differ considerably from MAF across breeds, with some alleles absent in all but one breed, in which they could be the major allele (see for example, SNP 78 and 189 in gene CfOR16HO4 and SNP 530 in gene CfOR0135). Other examples are provided by SNP 294, 518 and 295 (of CfOR0297, CfOR5413 and CfOR10F04 respectively), for which the minor alleles at the whole population level are the major alleles in one breed (Table 2).

**Table 2:** Overrepresentation of minor alleles in specific breeds.

**OR name**	**SNP**	**Position**	**GSD**	**BM**	**ESS**	**Grey**	**LR**	**Pek**	**6 breeds**
**CfOR16H04**	T/C	78	**0.562**	0	0	0	0	0	0.096
**CfOR16H04**	T/A	189	0	0	0	0	0	**0.562**	0.096
**CfOR0135**	T/C	530	0	0	0	**0.688**	0	0	0.115
**CfOR0297**	A/G	294	0.062	0.125	0.125	0	0.125	**0.688**	0.188
**CfOR5413**	A/G	518	0	0.062	0	0	0.125	**0.75**	0.156
**CfOR10F04**	G/A	295	**0.625**	0.125	0	0	0	0	0.125

Numbers correspond to the frequency of the allele identified as the minor allele in the whole population. The second allele in the SNP column is always the minor allele in the whole population. As expected, the minor allele at the whole population level is also the minor allele in most breeds. Exceptions in which the minor allele at the whole population level is the major allele in one breed are indicated in bold typeface. This table is a subset of additional file 3.

We found that 193 of the 732 SNP (26.4%) identified in this study were restricted to a single breed and that their breed distribution differed significantly (chi^2^, P <10^-3^), with 10 private SNP for German Shepherd Dog, 26 for Belgian Malinois, 47 for English Springer Spaniel, 18 for Greyhound, 8 for Labrador Retriever and 84 for Pekingese. Conversely, 199 SNP (27.2%) were common to all breeds, whereas 79 were common to two breeds and 50 were common to three breeds (Tables 3, 4 and 5).

**Table 3:** SNP distribution within breeds.

**SNP number**	**Breeds number**
199	6
120	5
91	4
50	3
79	2
193	1

**Table 4:** Number of SNP shared by different pairs of breeds.

	**BM**	**ESS**	**Grey**	**LR**	**Pek**
**GSD**	9	3	0	4	1
**BM**		14	1	3	19
**ESS**			0	8	4
**Grey**				7	5
**LR**					1

**Table 5:** Number of SNP shared by different trios of breeds.

**Breeds triplets**	**SNP number**
**GSD/BM/LR**	9
**GSD/BM/Pek**	2
**GSD/BM/Grey**	1
**GSD/ESS/Pek**	1
**GSD/BM/ESS**	1
**BM/ESS/LR**	9
**BM/Grey/Pek**	3
**BM/LR/Pek**	5
**BM/Grey/LR**	1
**BM/ESS/Grey**	1
**ESS/Grey/LR**	8
**ESS/LR/Pek**	3
**Grey/LR/Pek**	5
**ESS/Grey/Pek**	1

Assuming, as is most likely, that each SNP appeared once in the evolutionary history of the dog, it follows that the 199 SNP common to all breeds probably arose before the separation of the six breeds and that most of the private SNP arose following breed separation. Based on the same rationale, it could be hypothesised that SNP common to two or three breeds arose before the separation of these breeds. Although the number of pairs in common differed significantly (chi^2^, P <10^-3^), the use of HCLUST [31] to construct dendrograms did not result in any clusters matching breed history. This is probably because the number of SNP common to pairs of breeds with a MAF > 10% was too small.

### Polymorphism level

**Figure 4:**
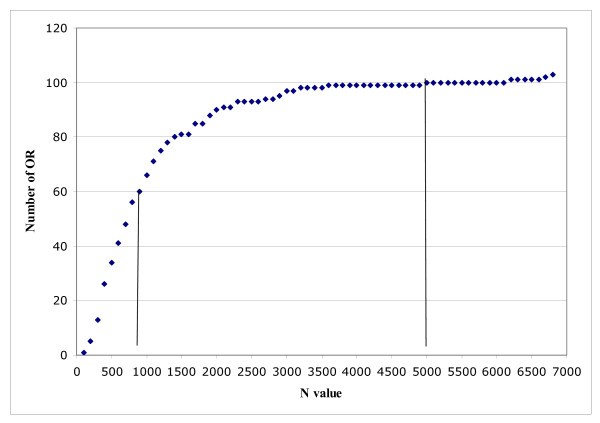
**Variability in OR gene polymorphism level**. Cumulative number of OR genes (y axis) plotted against N values (x axis). The graph shows that more than 50% of OR genes are highly polymorphic, with an N value even smaller than that for anonymous sequences (see Table 6), whereas ~10% are barely polymorphic (N > 5000) (see additional file 2). Note that six OR genes with a very high N value were off-scale and were not plotted on this graph.

Nucleotide polymorphism level reflects the number of differences between two sequences. It can be represented by N, the mean distance, expressed in nucleotides, between two SNP. OR genes are generally highly polymorphic, but the distribution of SNP is far from even (Figure 4). CfOR0034, in which 22 SNP were detected, was the most polymorphic OR gene studied, with an N of 98 for the whole population, ranging from 89 for Pekingese to 293 for German Shepherd Dog (see additional file 2). At the other extreme, CfOR08C09 and CfOR0525 were the least polymorphic genes after the four genes with no SNP (CfOR16F03, CfOR0317, CfOR0166 and CfOR0154). CfOR08C09 has one SNP, detected only once, in one Pekingese. This would give a theoretical N value of 7920 for Pekingese and 47520 for the whole population. Another example is provided by CfOR0525, for which we found 2 SNP. Each of these two SNP was detected only once, in two different Belgian Malinois, and one of these two SNP was detected in three English Springer Spaniels and two Labrador Retrievers (data not shown). This gives N values of 3780, 2908 and 4050, respectively, for these three breeds (see additional file 2).

Calculation, at the whole-population level, of N for the 109 OR genes gave a mean value of 577. Comparison at the breed level indicated that the English Springer Spaniel was the most polymorphic breed, with an N value of 594, whereas the German Shepherd Dog was the least polymorphic breed, with an N value of 926 (chi^2^, P < 10^-3^) (Table 6).

**Table 6:** Mean N values for OR genes and other sequences.

	**Total size (bp)**	**SNP number**	**GSD**	**BM**	**ESS**	**Grey**	**LR**	**Pek**	**6 breeds**	**Box**
**109 OR genes**	103762	733	926	617	594	778	634	628	577	ND
**27 OR genes**	25545	214	746	577	521	656	552	615	515	1728
**Exons**	3685	3	29480	29480	9213	5669	10284	8189	8631	ND
**Introns**	4766	10	2948	2487	1993	2334	2183	2373	1992	ND
**Intergenic sequences**	18716	97	864	943	848	735	878	863	732	ND

Mean N values were calculated as indicated in the Method section for the complete set of 109 OR genes and for a subset of 27 OR genes analysed in Boxer. Mean N values for exons (outside OR genes), introns (outside OR genes) and for anonymous intergenic sequences were calculated on the 48 dogs cohort. ND: not determined.

Only 27 OR genes were analysed in Boxer, and we obtained an N value of 1728. We therefore wondered whether the large differences in N values between the other six breeds and Boxer were due to the 27 OR genes selected for study in Boxer or whether they reflected a truly lower level of polymorphism in Boxer. However the N values for these same 27 OR genes calculated for each of the six breeds were not statistically different (Mann-Whitney test) from those calculated for the whole set of 109 OR genes (Table 6). This last finding ruled out the possibility of a bias due to the sampling of this subset of OR genes and indicated that the level of polymorphism really was lower for Boxer OR genes – this finding is relevant to the choice of the Boxer Tasha DNA sample (less polymorphic than the other DNA samples tested) for determination of the dog genome sequence [23].

We compared the level of OR gene polymorphism with that of non-coding regions and coding regions devoid of OR, by sequencing a series of exons, introns (only regions close to splice sites) and intergenic sequences with no known coding function. We obtained N values of 8631 for exons, 1992 for introns and 732 for anonymous intergenic sequences (Table 6). These values are consistent with previous reports [23]. A comparison of these values indicates that the coding regions of OR genes are more polymorphic than most exon sequences and more polymorphic than the non-coding DNA (chi^2^, P <10^-3^).

In a similar study, Sutter *et al. *[26] sequenced five non-coding regions of the dog genome in a cohort of 95 dogs of five breeds and detected 201 SNP and 19 indels. These results, indicating a lower level of genetic diversity than that observed in OR genes, confirm the high level of genetic diversity of the OR coding exons. The isolated OR genes and genes belonging to small clusters analysed in this study were overrepresented among the 109 OR genes as with respect to their presence in the whole repertoire. As these OR genes tended to be less polymorphic than the OR genes from large clusters, their presence increases the value of N, and the actual difference between OR genes and intergenic sequences should thus be even greater.

### Ka/Ks and protein sequence polymorphism

We noted that 152 of the 732 SNP identified within the 109 OR genes led to pseudoalleles (alleles with an interrupted coding frame). Theoretical translation of intact OR genes showed that 307 of the remaining 580 SNP were silent mutations. Of the 273 missense mutations (47% of the total), 130 would result in the incorporation of an amino acid of a different chemical group (Table 7).

**Table 7:** Distribution of the 580 SNP (307 silent and 273 missense) between the extracellular (EC), transmembrane (TM) and intracellular (IC) domains.

**Domain name**	**Number of missense SNP with AA group change**	**Number of missense SNP without AA group change**	**Total number of missense SNP**	**Total number of silent SNP**
**EC1**	12	7	19	20
**TM1**	7	17	24	21
**IC1**	8	3	11	8
**TM2**	7	8	15	27
**EC2**	5	8	13	17
**TM3**	4	5	9	7
**IC2**	9	5	14	15
**TM4**	7	16	23	20
**EC3**	13	12	25	45
**TM5**	12	6	18	21
**IC3**	20	11	31	16
**TM6**	5	15	20	35
**EC4**	5	9	14	13
**TM7**	4	12	16	22
**IC4**	12	9	21	20
**Total**	**130**	**143**	**273**	**307**

Calculation of the Ka/Ks ratio, where Ka is the number of non-synonymous substitutions (missense mutations) per non-synonymous site and Ks is the number of synonymous substitutions (silent mutations) per synonymous site between two closely related species, is the traditional method of assessing the strength of selection affecting proteins during evolution. In a recent study, it was shown that the A/S ratio calculated from the SNP content of the human genome is equivalent to the Ka/Ks ratio for the assessment of selective pressure [32].

Using the SNP detected in this study, a Ka/Ks value of 0.37 was obtained for the 95 OR genes analysed here (109 minus pseudogenes and non-polymorphic genes). Similar values were obtained at the breed level (from 0.31 for Labrador Retriever to 0.37 for Pekingese). A Ka/Ks value of 0.098 has been reported for a large set (n = 13,816) of canine genes [23]. Comparison of these two values (0.37 and 0.098) indicates an absence of strong selective constraint, resulting in greater diversification for the OR genes, as already observed for a small subset of human and chimpanzee OR genes and for the gene encoding the human bitter taste receptor, than for most other genes [33,34]. As isolated OR genes tended to be less polymorphic than OR within large clusters, we wondered whether the Ka/Ks ratio might differ with cluster size. A Pearson correlation test on the 95 OR genes analysed (all OR genes minus the pseudogenes and genes devoid of SNP) gave a value of -0.05059135, indicating this was not the case. Similarly the Ka/Ks values of the 11 OR genes within small clusters (≤ 5 OR genes) and the values for the 15 OR genes present in the largest cluster (243 OR genes) were not significantly different (Student's *t*-test P = 0.78).

We also analysed the distribution of SNP within codon positions and found that 161, 130 and 289 of the 580 SNP were located at the first, second and third codon positions, respectively. This distribution, with 50% of mutations affecting one of the first two positions, at which nearly all mutations induce an amino-acid change, and 50% affecting the third position, at which half of all mutations induce an amino-acid change, is consistent with many mutations (75%) randomly affecting the DNA sequence being retained and not counter-selected.

SNP were found throughout the OR gene sequences, resulting in amino-acid substitutions evenly distributed along the length of corresponding proteins, in the transmembrane, inner and outer parts of the receptors (Table 7).

However, if we take into account the respective sizes of the various domains, the number of missense mutations is significantly larger in intracellular (IC) than in extracellular (EC) and transmembrane (TM) domains (chi^2^, P < 10^-3^), whereas the number of silent mutations does not appear to differ significantly between domains (chi^2^, P > 0.7). These results were obtained for the whole set of data considered together, or when OR belonging to small clusters (≤ 5 OR genes) and OR belonging to the large cluster (243 OR genes) were considered independently. This indicates the existence of stronger selective pressure to maintain the structural conformation of the parts of the OR related to ligand binding (TM 3, TM5 and EC3 [9]) than to maintain the structure of the part of the protein involved in signal transduction and processing. This finding, which conflicts with those of Buck and Axel [1], should be interpreted taking into account the fact that we compared the sequences of the same gene in different breeds, whereas Buck and Axel [1] compared paralogous OR genes from a single rat and thus compared OR with different binding properties. It would thus be of interest to determine whether the amino-acid changes within IC domains affect the efficiency of the transduction pathway and, in turn, odorant sensing properties. The distributions of missense and silent mutations for the 136 SNP present in only one breed (private SNP) and for the 168 SNP shared by all six breeds indicate a significant bias, with missense mutations more frequent among private SNP (chi^2^, P < 10^-2^), suggesting selection pressure related to breeding practices.

We used the CORP program to determine the effects, if any, of the 273 missense mutations [35]. Of the 83 OR genes with missense mutation(s), 44 conserved the same Ψ_L _value, whereas changes < 0.3 were observed for 20 OR and changes > 0.3 for 19 OR. Variations of this type were also associated with higher or lower functionality as defined by the CORP program. As concerns a putative decrease in functionality, only 14 of the 273 SNP leading to an amino acid changes affect the 22 most highly conserved positions [9]. In addition, five missense mutations involved the arginine of the MAYDRY conserved motif.

### Pseudogene formation

Mammalian OR repertoires contain a large number of pseudogenes – up to 60% for the human repertoire and around 20% for the rodent and dog OR repertoires [4-8]. These pseudogenes are not retrogenes and have resulted from nonsense mutations or short indels occurring during evolution. Of the 109 OR genes analysed in this study, seven were strictly pseudogenes, 86 were intact in all breeds and 16 OR genes had both intact and interrupted ORF (pseudoallele). In each breed, a subset of 10 to 13 of these 16 OR have been identified as having one or more pseudoalleles (Table 8). The frequency of SNP closing the frame varies across breeds (Table 8). For example, CfOR08G02 has an SNP 360 (360 indicates the nucleotide position) that closes the frame. It is present in all six breeds, but at very different frequencies: 0.812 in German Shepherd Dog, 0.375 in Belgian Malinois, 0.125 in English Springer Spaniel, 0.188 in Greyhound, 0.438 in Labrador Retriever and 0.062 in Pekingese. Other examples, such as the SNP 362 of CfOR14A11 or SNP1 of CfOR12F06, are provided in Table 8. More extreme distributions exist, with SNP closing the frame in one or more breeds, but not all, such as the SNP 84 of CfOR0821 or SNP 49 of CfOR0401, which close the frame only in Pekingese and English Springer Spaniel, respectively. Genotype analysis (data not shown) indicates that the distribution within breeds is not homogeneous, with dogs having zero, one or two alleles with an interrupted ORF. These results indicate that the status of a gene as active or inactive (pseudogene) does not necessarily apply to the whole dog population, depending instead upon breed or even the individual dog. These observations suggest that pseudogene formation is still an active process, as previously reported [18,36], related to the acceptance of a large proportion of mutational events to the probable continuing diversification of the OR repertoire – the risk attached to deleterious mutations being counter-balanced by the highly combinatory nature of the OR repertoire [37,38], partly accounted for by gene redundancy.

**Table 8:** Pseudoallele frequency (PAF).

**OR name**	**SNP type**	**SNP position**	**GSD**	**BM**	**ESS**	**Grey**	**LR**	**Pek**	**6 breeds**
**CfOR0004**	indel	351	0.062	0.562	0.188	0.438	0.125	0.062	0.24
	NS	823	0.688	0.312	0.375	0.438	0.562	0.562	0.49
	indel	468	0.188	0.062	0	0	0	0	0.042
**CfOR0043**	NS	737	0.812	0.875	0.812	1	0.938	0.438	0.812
**CfOR0135**	indel	27	0	0	0.062	0	0	0	0.01
**CfOR0180**	indel	89	0	0.062	0.062	0	0	0	0.021
**CfOR0401**	NS	49	0	0	0.062	0	0	0	0.01
**CfOR0438**	indel	20	0.625	1	0.875	0.812	0.562	0.938	0.802
**CfOR04C05**	indel	70	0	0	0	0	0	0.125	0.021
**CfOR0519**	NS	306	1	1	1	0.938	1	1	0.99
	indel	289	0.062	0	0	0	0.188	0	0.042
	indel	536	0	0	0	0.188	0	0.062	0.042
**CfOR0565**	NS	790	0	0.062	0.062	0	0	0	0.021
**CfOR0821**	NS	84	0	0	0	0	0	0.125	0.021
**CfOR08G02**	NS	360	0.812	0.375	0.125	0.188	0.438	0.062	0.333
**CfOR12F06**	NS	1	0.875	0.5	0.375	0.25	0.75	0.375	0.521
**CfOR14A11**	indel	362	0.125	0.75	0.375	0.75	1	0.125	0.521
	indel	204	0	0.125	0	0	0	0.25	0.062
**CfOR16C11**	indel	633	0.125	0.188	0.438	0.25	0.188	0	0.198
**CfOR3109**	indel	89	1	1	0.625	0.428	0.75	1	0.798
	indel	306	0	0	0	0.143	0	0	0.024
**CfOR5912**	NS	658	0.25	0.188	0	0.188	0.312	0.062	0.167

PAF was calculated independently for each breed and for the whole population. Pseudoallele distribution varies considerably between breeds, with some pseudoalleles present in all breeds and some in only one or a few breeds: 9 are common to all breeds, 5 are found in only one breed and 8 are shared by two to five breeds, giving 10, 12, 13, 10, 10 and 11 OR pseudogenes for GSD, BM, ESS, Grey, LR and Pek, respectively. NS: nonsense, indel: insertion/deletion.

### Haplotype structures and distribution

**Figure 5:**
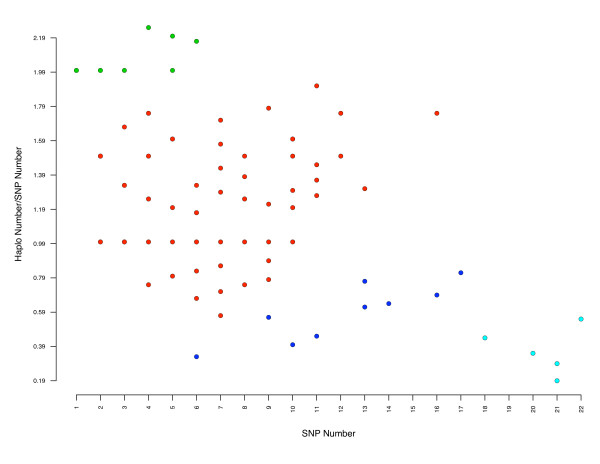
**Relationship between SNP and haplotype number**. Distances between points were calculated with R software (maximum distances) [43] and used to cluster OR genes. With k = 4, a group of 5 OR genes (in light blue) with a large number of SNP but a small number of haplotypes was identified, together with a group of 11 OR genes (in green) with a large number of haplotypes and a small number of SNP. We excluded from this last group the 4 OR genes with only one SNP and 2 haplotypes. Note that an individual point may correspond to more than one OR gene.

We used the Fast Phase algorithm [27] to identify a total of 809 haplotype structures for all OR genes with more than two SNP (see additional file 4). We found that the mean number of haplotypes per gene and per breed varied between 2.83 for German Shepherd Dog and 3.73 for English Springer Spaniel. Not surprisingly, the number of haplotypes per gene increased with the number of SNP. However this relationship is not simple and many exceptions were noted. We plotted the haplotype/SNP number ratio against the number of SNP (Figure 5). We calculated the Manhattan distances between the points and generated four groups of OR genes by agglomerative hierarchical clustering, with the two extreme groups having 11 OR and 5 OR genes. As examples of these two extreme groups, CfOR12A07 has 4 SNP and 11 haplotypes and DOPRH07 has 21 SNP and 4 haplotypes (see additional file 4).

The existence of the two extreme groups (Figure 5) suggests two different evolutionary processes. However, comparisons of gene status (family, subfamily, CFA position, cluster position for OR genes belonging to these two extreme groups) identified no specific feature.

As pointed out above, most of the SNP common to all six breeds had different MAF. Not surprisingly, this leads to very different haplotype patterns in different breeds, with some breed-specific haplotypes, such as the GCAGAGGTAAT haplotype (CfOR5413), which was found in 11 of the 16 Pekingese haplotypes but was absent from the other breeds (see additional file 4).

In total, we identified 332 breed-specific haplotypes (41%). Many (205) were found only once, but some (38) accounted for 25% or more of the 16 possibilities per OR gene per breed and might even be the most frequent haplotype in the breed concerned (Table 9). The combination of a small number of haplotypes may result, for each breed, in a haplotype signature. This signature could be used to certify that a given animal does or does not belong to a specific breed, based on the analyses of limited numbers of OR genes. For example, the haplotype structure of CfOR0050 and CfOR16H04, deduced from the analysis of 11 SNP, would be sufficient to identify a dog as a German Shepherd Dog.

**Table 9:** Number of breed-specific haplotypes and number of times represented.

	**GSD**	**BM**	**ESS**	**Grey**	**LR**	**Pek**	**Time fold/breed**
	11	41	43	24	25	61	1
	8	14	13	10	9	12	2
	6	2	2	1	4	8	3
	3	2	7	2	4	10	4
	1	0	0	0	0	2	5
	0	0	0	0	0	2	6
	1	0	0	2	0	1	9
	0	0	0	0	0	1	11
**Total number of breed-specific haplotypes**	**30**	**59**	**65**	**39**	**42**	**97**	

There are 11 specific haplotypes, each present once, in GSD and 1 specific haplotype present 11 times in Pek.

### Linkage disequilibrium (LD)

Linkage disequilibrium indicates an association between two polymorphic markers, for which pairs of alleles are inherited together. Previous studies have shown that dogs display higher levels of LD than humans. However, LD has also been shown to be heterogeneous, with alternating genomic long and short regions of LD [23]. This pattern of alternating long and short LD regions, which differs between breeds, has been attributed to the history of the dog population, which has been characterised by two bottlenecks and expansion periods [23,26]. We investigated the evolution of the OR gene repertoire by calculating LD both within and between OR genes.

#### LD within OR genes

All pairs of SNP (MAF > 0.05) within each OR were used to calculate the mean r^2 ^per breed – range of 0.52 for Pekingese to 0.70 for German Shepherd Dog, with a mean of 0.33 for the whole population (Table 10). These values indicate (1) that the extent of LD for OR genes is one tenth the mean extent of LD previously reported [23]; (2) the lower r^2 ^value (0.33) obtained for the whole population than for individual breeds is consistent with greater homogeneity within breeds. This low LD value indicates that SNP alleles within individual OR genes are not inherited as a block and suggests an ongoing gene conversion process potentially generating many OR genes with higher levels of polymorphism than the bulk DNA [39,40].

**Table 10:** Intra OR r^2 ^values.

	**GSD**	**BM**	**ESS**	**Grey**	**LR**	**Pek**	**6 breeds**
**intra-OR r**^2^	0.698	0.552	0.559	0.652	0.572	0.525	0.334
**SNP pairs number**	1027	1701	1557	1181	1447	1903	3368

r^2 ^values were calculated for pairs of SNP (MAF > 0.05) located within coding OR exons.

#### LD within OR clusters

A number of the sequenced OR genes corresponded to several clusters between 104 kb and 182 kb in size (see clusters description in additional file 5). We first retrieved SNP with a MAF > 0.2 and calculated D' values for each pair of SNP. The percentage of SNP pairs with a D' value > 0.8 varied from 38 to 66% for the five different clusters analysed within the whole population (Table 11). Contrasting results were obtained for analyses within breeds. For example, Belgian Malinois and Greyhound, in cluster 03, were weakly polymorphic and no LD value was calculated, whereas, for German Shepherd Dog and Labrador Retriever, 100% of SNP pairs had a D' value > 0.8 and, in Pekingese, only 58% of SNP pairs had a D' value > 0.8. These results indicate that the constraints imposed on OR cluster evolution are not identically distributed in the different breeds. The LD value calculated per breed was also higher than that calculated for the whole cohort (Table 11). This result contrasts with the findings of Sutter *et al*. [26], showing that the LD value calculated at the whole-population level for regions devoid of OR genes was similar to that obtained for individual breeds. However, our result is consistent with that reported by Menashe *et al*. [41] for the analysis of a human OR cluster in different populations.

**Table 11:** Percentage of SNP pairs with a D' value > 0.8.

	**GSD**	**BM**	**ESS**	**Grey**	**LR**	**Pek**	**6 breeds**
**cluster 01**	ND	ND	75%	89%	82%	66%	38%
**cluster 02**	64%	79%	78%	ND	64%	ND	66%
**cluster 03**	100%	ND	78%	ND	100%	58%	55%
**cluster 04**	89%	76%	69%	87%	61%	ND	52%
**cluster 05**	ND	73%	82%	ND	64%	81%	45%
**5 clusters**	**94%**	**75%**	**78%**	**85%**	**70%**	**78%**	**48%**

These values were identified within 5 clusters of 104 to 182 kb (these clusters are described in additionnal file 5). ND: not determined (too few SNP pairs).

## Conclusion

We have shown here that overall OR gene diversity is very high, with a mean distance (N) between SNP of 577 nt, slightly less than that calculated for non-coding sequences and much shorter than the distances calculated for exon sequences. However, this diversity is not uniformly distributed, some OR genes having few or no SNP, whereas others may have as many as 22 SNP in their coding sequence. In addition, individual OR genes may be highly polymorphic in one or a few breeds and devoid of SNP in other breeds. Thus, the overall level of polymorphism was found to differ between breeds, with a mean distance of 628 for the Pekingese and 926 for German Shepherd Dog. An even higher N value was calculated for the Boxer, consistent with previous suggestions of a lower level of genetic diversity in this breed [23].

As the presence of different alleles of specific OR genes has been shown to affect the perception of isovaleric acid and androstenone in humans [16,17], this OR genetic diversity, with 47% of SNP leading to missense mutations, should clearly affect the odorant sensing capabilities of dogs. However, as the ligands of most of these OR are unknown, it is not possible yet to correlate the OR genetic polymorphism with variation in odorant perception. The level of polymorphism for about 50% of the OR genes was found to be higher than that for anonymous sequences, for which all, or almost all mutations arising during DNA replication are probably conserved. As there is no evidence to suggest that replication is itself defective, another mechanism, such as gene conversion, should be considered to account for this higher level of polymorphism, as suggested by the low LD values calculated within OR genes.

This process, which is of great importance in maintaining sequence homogeneity in genes with multiple copies, such as histone genes, has been proposed as a mechanism guiding the evolution of paralogous OR genes [40,42]. We suggest that this mechanism may be involved in the accumulation of SNP, although some of these mutations may lead to a less functional OR or may be nonsense mutations.

The accumulation of mutations diversifying OR amino-acid sequences may have two opposite effects that must be balanced: an increase in odour pattern recognition and the risk of a loss of function. Such losses of function do occur, as indicated by the ongoing pseudogenisation observed. However, the risk of losing the ability to sense a particular odorant is minimized by the highly combinatory code [37,38]. Nonetheless, not all OR genes are polymorphic, and up to 22% of the OR genes in an individual breed may be entirely non-polymorphic. This raises the possibility that these non-polymorphic OR may be involved in recognising odorants of particular importance or may have a unique binding specificity not shared by other OR. Finally, we observed that, for each breed studied, it was possible to define specific haplotypes for a number of OR genes characteristic of the breed, which could be used as a genetic signature to determine whether or not a particular dog belongs to a particular breed.

## Authors' contributions

SR carried out molecular genetic studies, interpreted the data and drafted the manuscript. ST carried out molecular genetic studies and participated in sequence alignment. MR carried out molecular genetic studies. AV participated in the statistical treatment of the data. SD determined the nucleotide sequences. CA provided the DNA samples. CH participated in the statistical treatment of the data. FG conceived, designed, coordinated the study and helped to draft the manuscript. All authors read and approved the final manuscript.

## Supplementary Material

Additional file 1 Analysed OR genes. Distribution of analysed OR genes within the canine OR repertoire. Click here for file

Additional file 2 OR names and numbers per cluster, SNP numbers and N values. OR name, size of OR genes clusters, number of SNP and N value for each breed. OR are classified by N value increasing. Click here for file

Additional file 3 MAF (minor allele frequency) of the 732 SNP. MAF (Minor Allele Frequency) of SNP within each breed and 6 breeds. Click here for file

Additional file 4 Haplotype structures. List of haplotypes within each breed. Click here for file

Additional file 5 Cluster description. Characteristics of OR clusters used for LD analysis. Click here for file
